# A Study Protocol for Applying User Participation and Co-Learning—Lessons Learned from the eBalance Project

**DOI:** 10.3390/ijerph14050512

**Published:** 2017-05-10

**Authors:** Anna Cristina Åberg, Kjartan Halvorsen, Ingrid From, Åsa Bergman Bruhn, Lars Oestreicher, Anita Melander-Wikman

**Affiliations:** 1 School of Education, Health and Social Studies, Dalarna University, SE-79188 Falun, Sweden; ifr@du.se (I.F.); asu@du.se (Å.B.B.); 2 Department of Public Health and Caring Sciences, Division of Geriatrics, Uppsala University, SE-75185 Uppsala, Sweden; 3 Department of Information Technologies, Division of Systems and Control, Uppsala University, SE-75105 Uppsala, Sweden; kjartan.halvorsen@it.uu.se; 4 Department of Information Technology, Division of Visual Information and Interaction, Uppsala University, SE-75105 Uppsala, Sweden; Lars.Oestreicher@it.uu.se; 5 Department of Health Sciences, Division of Health and Rehab, Luleå University of Technology, SE-97187 Luleå, Sweden; Anita.Melander-Wikman@ltu.se

**Keywords:** user participation, reflective practise, action research, co-learning, implementation

## Abstract

The eBalance project is based on the idea that serious exergames—i.e., computer gaming systems with an interface that requires physical exertion to play—that are well adapted to users, can become a substantial part of a solution to recognized problems of insufficient engagement in fall-prevention exercise and the high levels of fall-related injuries among older people. This project is carried out as a collaboration between eight older people who have an interest in balance training and met the inclusion criteria of independence in personal activities of daily living, access to and basic knowledge of a computer, four staff working with the rehabilitation of older adults, and an interdisciplinary group of six research coordinators covering the areas of geriatric care and rehabilitation, as well as information technology and computer science. This paper describes the study protocol of the project’s initial phase which aims to develop a working partnership with potential users of fall-prevention exergames, including its conceptual underpinnings. The qualitative methodology was inspired by an ethnographical approach implying combining methods that allowed the design to evolve through the study based on the participants’ reflections. A participatory and appreciative action and reflection (PAAR) approach, accompanied by inquiries inspired by the Normalization Process Theory (NPT) was used in interactive workshops, including exergame testing, and between workshop activities. Data were collected through audio recordings, photos, and different types of written documentation. The findings provide a description of the methodology thus developed and applied. They display a methodology that can be useful for the design and development of care service and innovations for older persons where user participation is in focus.

## 1. Introduction 

The rationale of the entire eBalance-project is based on the idea that serious exergames, i.e., digital gaming systems with an interface that requires physical exertion to play [1], that have a health related aim beyond pure pleasure, and are well adapted to users, can become a substantial part of a solution to the well-recognized problems of low insufficient engagement in fall-prevention exercise and the high levels of fall-related injuries among older people. This paper focuses on the study protocol of the initial phase of the eBalance project, which aims to develop a working partnership with potential users of fall-prevention exergames. The protocol describes the methodology developed and applied for this purpose, including its theory and underpinnings, which are discussed. 

Decreased balance control has been shown to be the most commonly occurring modifiable fall risk factor [2,3,4]. Convincing evidence has shown that balance exercises are the single most effective fall-prevention intervention among community-dwelling older adults [5,6,7,8,9]. However, in a randomised fall-prevention study, one-third of older people (≥65 years) who had fallen at least once during the previous year refused physical therapy when it was recommended [10]. Reasons given included: difficulty travelling, concern about cost, and disbelief in the efficacy of the interventions. Moreover, interviews about older people’s views on fall prevention conducted with people aged 68–93 years in six European countries [11] revealed that their preferences were similar in all countries, and in line with the above results. Barriers to participation included denial of fall risk, opinions that fall prevention was unnecessary, and several practical barriers concerning transportation, effort, and cost, as well as a dislike of group activities. On the other hand, a more recent review of the perspectives of older people (≥65 years) [12] resulted in the identification of four themes considered important for participation in fall-prevention programs; (i) Influence of participants and program characteristics, (ii) personal relevance and preferences, (iii) maintaining autonomy and independence, and (iv) support and access. The importance of understanding older people’s interpretation of fall-prevention messages and its integration into daily life were stressed. Another review exploring the same issue among the same age group of community dwellers, with the addition of healthcare professionals [13], highlighted the complexity and multifactorial characteristic of barriers and facilitators for fall-prevention intervention. Recommendations for the implementation of fall-prevention based on these results include considerations of: (i) practicalities, (ii) adaptation for social and cultural influences, and (iii) psychosocial considerations including transforming identities (with the influence of associations to “risk of falling”) and definition of the fall-prevention expert (including healthcare professionals and significant others). 

The problem of insufficient fall-prevention balance training appears, hence, to be multifaceted and comprises both attitudes among older individuals in need of such training and insufficient provision of easily assessable guidance and support that is adapted to personal needs and preferences. It may be possible to reduce the magnitude of this problem by introducing appropriate exergames that are designed to meet the requirements of those in need of the exercise and by supplying staff to assist them. In a recent report from the European Union (EU), the “lack of participation by citizens, patients, care givers, and healthcare professionals in the design, implementation and evaluation” ([14], p. 4) of new care systems and innovations for older people was pointed out as a serious problem that jeopardises better care quality and sustainable and efficient health and care delivery systems in Europe. 

The use of exergames for balance training has been described as highly enjoyable, and therefore motivating. It has also been described as holding promising opportunities for balance improvement in older adults, which in turn may lead to fall prevention [15,16,17,18,19].

Identified advantages of exergames when compared to ordinary exercises include their ability to prevent monotony and boredom, increase motivation, provide immediate feedback, and allow dual-task training [18]. However, data on fall prevention outcomes are still lacking [18] and the methodological variation between studies and the overall modest study quality has been emphasised [17,19]. In order to become truly successful tools for improving balance performance, new appropriate games that are tailored specifically for older people and that are safe and easy to use are needed [19]. Moreover, comparing differences in games and in game movement requirements should provide valuable insight into designs for movement quality [20]. 

A review [21] aimed at elucidating older adults’ perceptions of different types of technologies for fall-prevention, showed that interactive functions and clear audio feedback, as well as feedback related to the level of performance, were perceived as very important for people’s motivation to use exergames. The conclusions drawn found that significant motivators from a user’s perspective included choice and control over the technology and acceptance of one’s need for the technology. Other motivators included usability and potential benefits from using the technologies, such as increased independence and social opportunities as well as improved user confidence and functions [22]. It has also been shown that among older Swedes, internet-based e-health services are anticipated to positively impact quality of life from a psychosocial perspective [23]. These latter results may be related to the fact that in Sweden, 89% of all individuals have access to a computer and the internet and the most rapid growth in the number of computer users is now seen in the older age groups [24]. The possibility of meeting psychosocial needs in combination with balance improvement may well be of particular importance in seeing that fall-prevention exercises are used over a longer period of time.

The eBalance project was planned with the overall aim of improved health and well-being among older people as a result of increased levels of balance exercising through the use of serious exergames. The aim of the initial sub-project, whose protocol is reported here, was to focus on optimization of the “implementation potential”, i.e., the probability of practicing such games becoming normalized and part of a work/all day routine [25]. For that reason, effort was initially put into the development of the study methodology in cooperation with users. To enable a clear impact of the users’ needs and recruitments in the project processes, achievement of joint interactive reflections and co-learning together with potential users of exergames for improved balance, in order to generate constructive study cooperation, became a central theme. Co-learning is hereby defined as interactive processes that can result in the involved individuals gaining new information and perspectives, which when accumulated can increase their knowledge and change their way of thinking.

The aim of the present paper is to describe the study protocol developed and used in the initial phase of the eBalance project, including its conceptual underpinnings and its focus on user participation and co-learning. 

## 2. Methods and Participants

The qualitative research design was inspired by pragmatic and ethnographic considerations [26], implying that the study was carried out in a non-laboratory and non-clinically everyday setting, combining methods that allowed the design to evolve through the study and focus on individuals reflections [27]. The current study was carried out as a collaboration between eight older people who have an interest in balance training and met the inclusion criteria of being independent in personal activities of daily living and having access to and basic knowledge of a computer, plus four staff working with the rehabilitation of older adults and an interdisciplinary group of six research coordinators covering the areas of geriatric care and rehabilitation, as well as information technology and computer science (Table 1). 

A Participatory and Appreciative Action and Reflection (PAAR) methodological approach was adopted [28] and the Normalization Process Theory (NPT) was applied as a guiding theory for inquiries directed towards factors of importance for a high level of implementation potential. According to NPT, it is possible to investigate the probability of a practice becoming normalized and routine, by assessing the potential of a practice to be implemented and the readiness of actors to accept it [29].

### 2.1. Ethical Considerations 

The project was approved by the Regional Ethical Review Board in Uppsala (Dnr. 2013/473). All data was treated confidentially and the integrity of the participants was protected, both during the research process and in the presentation of the results.

### 2.2. The eBalance-Technology

**Figure 1:**
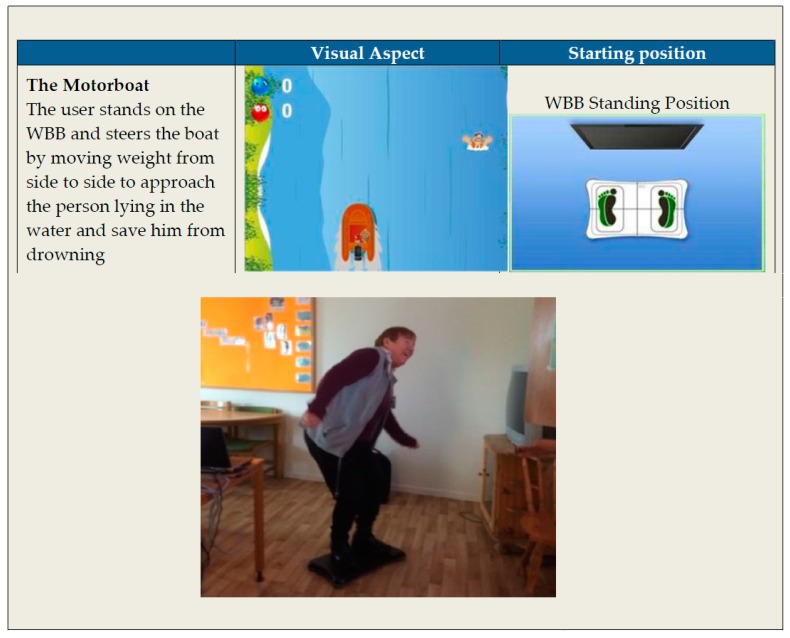
An example of a serious exergame in play using a Wii Balance Board (WBB).

The exercise application for serious games that the participants physically interacted with as part of the workshops (WSs) consists of available gaming technology and an ordinary computer (Figure 1).

It was used to provide exercises in the form of prototypes of serious balance exergames that are designed to get users to move their weight in different directions.

We used two different technologies: the Wii Balance Board (Nintendo Co., Ltd., Kyoto, Japan), which is a pressure-sensitive force plate, and the PrimeSense 3D sensor (PrimeSense, Tel Aviv, Israel), which uses a camera-based sensor. The latter sensor is included in the Microsoft Kinect product. Our intention was to start from the basis of existing software specifically designed for rehabilitation. We had licenced access to two different systems: The first was developed for the Wii Balance Board (WBB) by the Universidad Polytecnica de Valencia, Spain [30] (see example Figure 1), and the second was a system developed for the PrimeSense sensor at the Centre for Rehabilitation Research Engineering at the University of South California, USA [31]. Both systems were developed for balance training. 

### 2.3. Methodological Approach and Theoretical Framework

We adopted the methodological approach of PAAR [28], to which the processes of collective working and appreciative knowledge sharing is central. Moreover, PAAR involves a strong focus being placed on factors that work well before identifying problems. Instead of only looking for problems that must be solved, this methodology emphasizes success and its root causes, so that success can be better understood and amplified. Hence, in the current study we emphasized what the user groups wanted more of and on what strengths and successes we could build together [28]. 

**Figure 2:**
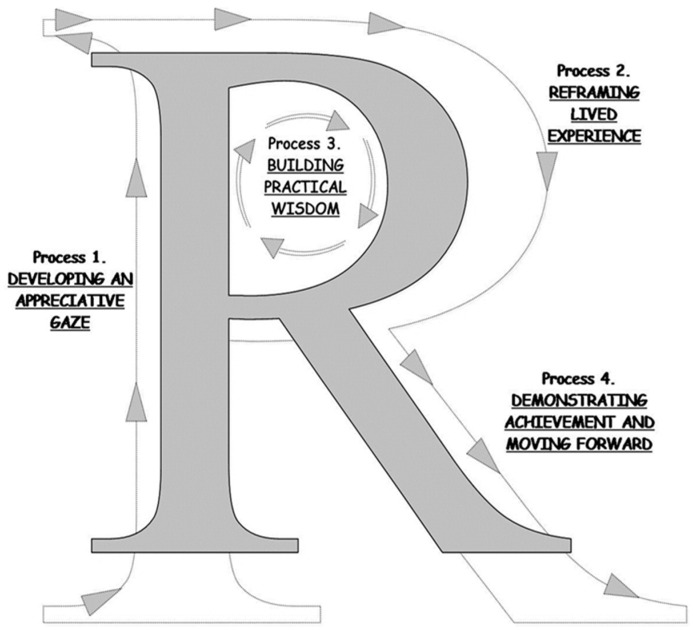
Participatory and appreciative action and reflection (PAAR) as a process [28], including the four supportive processes: (I) Developing an appreciative gaze, (II) Reframing lived experiences, (III) Building practical wisdom, and (IV) Demonstrating Achievement. **Process (1)** *Developing an appreciative gaze*, implying a start with appreciation of our own and others’ gifts, and open-minded considerations of what is happening in meetings and interactions, to support human flourishing and well-being in current thinking and practise. **Process (2)** *Reframing lived experience* is an important and often difficult process, aspiring to build a better future from aspects of the positive present. Its essence is about viewing problems or challenges in a creative way to seek new approaches. **Process (3)** *Building practical wisdom* is about seeking to generate new practical and meaningful insights and improved actions, and not only to produce refinement of what is already known. A central feature of this process is knowledge sharing and the generation of collective wisdom, to create something that is greater than any individual could build alone. **Process (4)** *Demonstrating achievement and moving forward* concerns the practical consequences of positive collective wisdom and action. The essence is about seeking future actions inspired by what the participants experience as valuable, celebrating, and sustaining.

There are four central and mutually supportive processes providing a practical description of the reflective approach which is essential for PAAR [28,32], see Figure 2.

Since, asking appreciative questions is a key part of the PAAR-type inquiry and a driving force for its reflective processes, for the formulation of such questions we, additionally, combined appreciative questions with ideas from the NPT [33]. The current protocol describes the cooperative working processes based on these approaches, between two potential user-groups of exergames, i.e., senior citizens (Seniors) and rehabilitation staff (Staff), and an interdisciplinary group of research coordinators (Coordinators) covering the areas of geriatric care and rehabilitation, as well as information technology and computer science (Table 1). In the present study, the PAAR-approach was used to direct the interactions for co-learning. So, the WS-sessions (see below) were designed with inspiration from the processes of the PAAR-type reflective inquiry, described by Ghaye et al. [28,32] (Figure 2).

The NPT was originally developed to provide support for complex interventions in health care [33]. This theory is concerned with explaining what work people do—or need to do—around implementing new practices, which is described in relation to the constructs [29].

(i) *Coherence*; i.e., the sense-making work that people do individually and collectively to operationalize new practices, (ii) *Cognitive Participation*; the relational work that people do to build and sustain a community of practice, (iii) *Collective Action*; the operational work that people do to enact a set of practices, and (iv) *Reflexive Monitoring*; the appraisal work that people do to assess and understand the ways that a new set of practices affect them and others. 

### 2.4. Data Collection and Analysis

Data were collected through audio recordings of the discussions during and after WSs, participant observations, photos of WS activities (in particular exercise tasks), and written documentation on post-it papers and flip boards as well as e-mailed reflections (by the users) and written summarising comments (by the Coordinators). All documentation, including the PowerPoint presentations by the Coordinators, was stored in a digital storage facility at the university that all Coordinators had access to. 

In line with the chosen methodology, data collection and analysis were carried out in parallel processes to enable methodology and inquiries to evolve throughout the study, with the goal of achieving co-learning and practical wisdom (Figure 2). The main methodological approach for this ongoing analysis was sharing reflections in different constellations of the study participants. For the current article, the first author (Anna Cristina Åberg) also went through and analysed all study material stored in the information technology (IT)-based storage, using ethnographic inspired content analysis [26,34] focusing on the description of the methodology developed and applied, i.e., the structure and content of the study activities and their distribution in time and space. This analysis was validated by parallel analysis of parts of the material carried out by IF (co-author and member of the Coordinator group) and by validating discussions with AMW and IF through the whole process of writing up. 

## 3. Interactive Procedures

The interactive work with the goal of co-learning among the participants, was initiated by the recruitment process, and later continued in five WSs and between WS activities, and finalized by a shared study report, all of which are presented below. 

The PAAR supportive processes [28,32] were applied in a cyclic manner of appreciation, action, reflection, and co-learning. The main study activities for the realization of PAAR are presented below, structured in the following Sections: Section 3.1 Organization and recruitment, Section 3.2 Interactive workshops, Section 3.3 Between WS activities, and Section 3.4 A shared study report.

### 3.1. Organisation and Recruitment 

The eBalance-project was led by two researchers in the area of geriatric physiotherapy (Anna Cristina Åberg, the first author) and information technology (Kjartan Halvorsen, the second author), respectively, with joint expertise in movement analysis including human balance control [35,36]. Since this project springs from ideas concerning the improved implementation of fall-prevention among older people, user participation was judged as essential from the very beginning. However, it did not involve users in the purely methodological considerations in the planning phase of the project. On the other hand, to prepare for constructive user-interaction, a scientific advisory group was formed, with expertise in PAAR used for the development of e-health solutions for older people, which included the last author of this manuscript (Anita Melander_Wikman). Additionally, an executive multi-disciplinary group of researchers and clinical experts in the areas of balance impairment and exercise, geriatric nursing, and human-computer interaction was established for the realisation of the current study in cooperation with the two project leaders; they all together constituted the group of Coordinators (Table 1). 

The Coordinators shared the driving initiatives for the study processes, including the recruitment of individuals representing the two potential user-groups of exergames, i.e., Seniors and Staff. Already in the initial contacts, effort was put into developing an appreciative gaze [37], characterizing the beginning of the PAAR-process (Figure 2). 

Recruitment was carried out in several steps (Table 2). The Seniors were invited to express their interest in participating in the study when they visited a Senior Health event in a small town in mid Sweden. For the recruitment of Staff, contact was made with responsible leaders working with the rehabilitation of older people living in the same local community, who approved participation during working hours for the Staff. In the next step, Seniors and Staff who had declared interest were invited to separate meetings, where the Coordinators informed them about the study; its background, purpose, and planned working methods. This was followed by a dialogue with potential participants focusing on their perspectives relating to the study subject and study participation per se.

Additionally, two versions of information leaflets, designed for Seniors and Staff, respectively, were distributed. They described the study and included contact information for a study contact person (one of the Coordinators). Those who decided immediately at the information meeting that they would like to participate filled in their name on a “list of interest”, while others were invited to make contact with the contact person if they later decided that they would like to be involved. This resulted in a list of interested people that included 18 Seniors and four Staff, from which a total of 8 Seniors were included as participants in the study as they met the inclusion criteria of being independent in personal daily living activities and had access to and basic knowledge of a computer. All four Staff were also included based on the fact that they were all working with the rehabilitation of older people.

Hence, eight Seniors, aged 67–85 and four Staff, of which three were physiotherapists and one was an occupational therapist, were included in the study. When asked about their motivation for joining the project, all participants expressed expectations that study participation would correspond to their different interests: The Seniors were motivated to participate by their interest in balance training, both due to experienced balance problems and engagement in exercise for older people. The Staff expected that participation would give them opportunities to develop useful knowledge in the area of fall-prevention. The common study interest of the Coordinators was to develop a deeper and more multifaceted understanding of the challenges related to their own area of expertise within the eBalance project (Table 1).

For the interactive and co-learning processes we strived to develop a reflective approach closely related to the idea of learning from practice. This was realized by blending theory and practise and viewing the users as active partners possessing valuable perspectives and knowledge and by encouraging active and interactive reflection to facilitate co-learning for all participants [38]. The users’ perspectives and expertise based on lived experiences and professional expertise were, accordingly, regarded as a valuable knowledge base with unique perspectives. Beside a long-lasting overall life experience, several of the Seniors had experiences of fall with or without related injuries. Some experienced problems with balance control, one of them due to a neurodegenerative disease, one lived with a relative with mobility problems including decreased balance control affecting daily living, and one had many years of experience as a fitness instructor for older adults. All the Staff worked with the rehabilitation of older people on a daily basis, which commonly involved the treatment of functional mobility limitations, often including some degree of decreased balance control. Their perspectives were based both on their working relationships with the older patients and on experiences from their working situation and organisation. The Coordinators’ perspectives were grounded in their areas of research and expertise, as mentioned above. Coordinators with a background as clinicians (physiotherapy and nursing) also had several years of experience of care work with older people, albeit several years ago. One responsibility for the Coordinators was to create opportunities and good conditions for the participants to jointly reflect upon, express, and share their experience or professional knowledge, or both, relating to the study aims. All participants were encouraged to engage in the study activities and to communicate their reflections and knowledge including ideas about improvements/modifications for fall-prevention exergames, as well as for the study interaction including the form of activities and their disposition in time and space. This involved sharing new experiences of study participation and exergaming, which were creatively reflected upon and reasoned around from our diverse perspectives, as a way of re-framing lived experiences and building practical wisdom in co-learning processes (Figure 2) as described in Section 3.

### 3.2. Interactive Workshops

The overall purposes of the WSs were to develop and test the study design as described above, and to scrutinize important factors for the improvement of implementation potential of fall-prevention exergames. Five thematic WSs were carried out at the Red Cross premises in the town where the participating user-groups lived and/or worked. Workshops 1–4 were held weekly for a four-week period, and WS5 was held seven weeks after WS4 to jointly summarize and evaluate what was learned.

Each WS had a specific theme (Table 3), i.e., WS1: Interaction, WS2: Fall-prevention, WS3: Balance exercise and IT-based support, WS4: User-needs and design requests, and finally WS5: What we have learned. Full day WSs (five hours including lunch) were modified to two-hour WSs (including coffee breaks) (Table 3). 

The WS activities were shaped from a starting point of rough blueprints, which mirrored the themes mentioned above. In an attempt to apply the process of PAAR, the content of the WSs was developed and reconsidered based on input from reflective discussions (around one hour) by the Coordinators at the end of each WS once the interactive part with the users was completed and they had left the room. As a result of these discussions, details were added and reformed based on an effort to understand and grasp essential viewpoints and needs from the perspectives of the user groups as well as making use of developmental ideas emerging during these discussions among the Coordinators, also taking into account input and ideas from participants between WSs.

Moreover, the NPT was operationalised by the formulation of questions (see Appendix A) related to the four constructs Coherence, Cognitive Participation, Collective Action, and Reflexive Monitoring [29] used as facilitators of reflective interactions around the WS themes and focusing on the overall aim of the eBalance study. These queries were additionally adapted to the context in question, using the Coordinators’ experience and literature-based knowledge. This resulted, for example, in questions on security being added as an ingredient related to the use of exergames. It should be noted that we intended to investigate implementation potential so several questions were directed towards the future and the users’ anticipated views on exergames. Since the NPT constructs Reflexive Monitoring, i.e., the appraisal work people do to assess and understand the ways that a new set of practices affect them and others around them, this construct was most obviously related to questions about the study interaction (Appendix A), as being an experienced area possible to retrospectively evaluate. However, our intention to demonstrate achievement and moving forward, when working with all these questions, culminated in WS5 with its theme; What we have learned. In addition, this process continued on after the last WS in the final phase, which involved the completion of a study report that was written for all the study participants (see Section 3.4 below).

### 3.3. Between Workshop Activities 

Between the WSs, possibilities for communication with and between the participants were initiated using e-mail communication. A project-specific Google discussion forum was also used between WSs for communication with the users about their views on issues relating to the study’s aim (Appendix A). These issues were introduced at WS1, 2, and 4, and presented as ‘homework’ issues to reflect and comment on. 

The NPT-inspired questions (Appendix A) were introduced and used with the intention of acquiring information useful for the forthcoming development and improvement of the implementation potential of a forthcoming eBalance exergame and related interactive implementation strategies. Thus, the purpose of the initial issue (WS1–2), *Meaningful activities in daily life*, was to start a dialogue inspiring the users to formulate joyful and important activities from their own perspectives, which could give a joined picture why good balance performance was positively desirable—besides reducing the risk of falling and suffering related injuries. For the following two homework issues (WS3–4 and WS4–5) we asked the users for their views on *IT-based support for balance exercises* and on *Interaction in the study*, respectively.

Between WSs, the Coordinators worked with post-WS reflections (described above), communication with users, planning, and preparation for forthcoming WSs. Between WS4 and WS5, the Coordinators also prepared a draft for a study report to share with the users, and to refine in collaboration with them. This refinement work, based on reflective inquiries, was initiated at WS5 and continued after it. 

### 3.4. A Shared Study Report

Inspired by the fourth process PAAR; Demonstrating achievement and moving forward (Figure 2) which corresponds to the NPT construct Reflexive monitoring [33], we completed a 24 page report, describing our joint work in ordinary non-scientific Swedish language. As indicated above, the basic ingredients of the report were presented, discussed, and reflected on at WS5. By sharing different stages of the drafted report with the users, they were given the opportunity to contribute and revise the content and validate the content of the final report including illustrations, i.e., photos from the WSs and graphic overviews of participants and findings. Finally, the finished version was printed and sent to all study participants approximately two month after WS5.

## 4. Discussion 

### 4.1. Combining the Appreciative Action and Reflection with Normalization Process Theory

We developed study methodology of reflective practise [38] by integrating theoretical and practical interactions, guided by PAAR as a style of research and NPT as a theoretical framework for the construction of questions used for the facilitation of reflections related to the study aims. The combination of the NPT and PAAR was experienced as easy to accomplish in an almost seamless way, probably because the ideas underpinning those concepts are derived from similar sources. The roots of both PAAR and NPT are to be found in sociological theory implying that people create meaning through interactions and reflections, which can also constitute a method for co-learning and shared knowledge creation. This implies a focus on the experiences that arise in social relations, with a common emphasis on human actions and meaning-making by interactions [39]. Accordingly, both PAAR and NPT contain elements of meaning or sense-making. In NPT, this is particularly evident in the construct Coherence, referring to different aspects of Sense-making work, that people do individually and collectively, when they are operationalizing some set of practices [33]. In relation to PAAR, it has been suggested that reflective practice may be a way of enhancing a sense of individual and collective meaning [37]. In this light, essential parts of dialogue including shared reflections that we applied, can be viewed as handling meaning in several ways; expressing their own interpretations of meaning, taking part of other perspectives of meaning related to the same issues and, hence, being provided with improved possibilities to reframe their own meanings in relation to new practical experiences (of the study work including exergames-testing), and others’ opinions and reflections. In other words, the methodology used facilitated shared definitions and re-definitions of meaningful factors and the creation of new knowledge and wisdom related to the study aims. 

### 4.2. Appreciative Inquiry, Interaction, and Participation Roles

Appreciative inquiry, the approach distinguishing PAAR from other forms of action research, is based on an assumption of the significance and inherent power of interaction [40]. According to Ghaye [32], the originator of PAAR, we can open up opportunities for new and different ways of action by changing the questions we ask and thereby changing the dialogue. In the current study, we put effort into appreciative formulations of the questions we interacted around, which were directed towards a high degree of implementation potential of both fall-prevention exergames and the methodology we developed in the course of the study. 

Effort was made to develop an appreciative gaze (Figure 2) [28], already in the stage of recruitment of study participants, by emphasizing qualities such as respect, compassion, objectivity, and trust, which are also required features of the type of exchange agreement or psychological contract [28,41] we intended to build with the participants of the user groups. Also, the homework task of describing *Meaningful activities in daily life*, was used for the purpose of developing an appreciative gaze and also for encouraging improvisations and narratives, opening up for a creative and sense-making dialogue, as suggested by Ghaye [32]. 

Development of an appreciative PAAR-related approach was sometimes challenging, in particular when technical problems arose, which impacted interaction. The fact that all the Coordinators, who had the main responsibility for the formulation of the study questions and for the facilitation of the interaction, had professional backgrounds in different traditionally problem-oriented scientific and clinical thinking, may also be considered. On the other hand, the effort for the development of an appreciative gaze was generally perceived as both important and developmental for the co-learning processes and for personal growth in the role as Coordinator. Reflections among the Coordinators at the end of each WS provided a necessary opportunity for reconsidering the interaction outcomes, for mutual support and, in line with the ethnographic study approach, the development of the design. These reflections brought about an awareness of the importance of framing each WS and the questions we discussed with clearly formulated aims that were reflected upon together with all participants to generate shared understanding and meaningfulness relating to our work. One possible reason for this appearing as an important need may be the different motives for participation (exposed in Table 1) mirroring quite diverse perspectives. Therefore, a procedure carried out at the start of every WS was initiated, which implied a presentation of the aim of the entire eBalance project complemented with the aim of the WS in question and joint reflections around this. Another related example is the interaction around practical tips and tests of balance exercises as a part of WS3, which were asked for by the user groups—in particular the Seniors. These activities became an incorporated meaning-making part of the development of the study methodology. 

Another lesson learned was that clarifying reflective discussions in an early stage of study accomplishment, about roles and responsibilities for participation was also essential, even though these roles need to be allowed to evolve in parallel with the study processes. Recurrent discussions concerning the Coordinators individual and shared responsibilities were necessary to allow such development and to avoid misinterpretations. Moreover, profound reflections for the development of mutual understandings and reframed meanings concerning each other’s use of language, definitions of concepts, and ways of expression, appeared as a necessary prerequisite for co-learning based on reciprocal understanding. It has been shown that communication between people in daily and caring situations often involves undiscovered misunderstandings [42], due to discrepancies concerning definitions and interpretations. There is a dilemma here in that as long as the discrepancies are not obvious, we are not aware of the problem. Not until we realise that we are not grasping the other party’s true messages and needs do we feel responsibility to view this as something we should consider. Communications that are built on hidden misinterpretations can therefore give a false impression of being satisfactory. Since the definitions that influence people’s behaviour are ongoing processes, it is possible, however, to re-frame and adjust the approaches, language, and behaviours in accordance with that re-framing [43], which was a central part of the co-learning PAAR-inspired processes [37].

The individuals of the user groups reported that they were overall very satisfied with their study involvement; they attended the WSs and showed active engagement, and also wished for a continuation at the end of the study. Their roles may anyhow be considered. The Staff participated as part of their ordinary work, and, hence kept their ordinary salary despite the time spent at the WSs. After finishing their study participation, some of them frequently took part in university activities, involving themselves in interaction and sharing of research results with the surrounding society, which in Sweden is imposed by the government as a third task for all universities in addition to high quality education and research [44]. We might speculate that their involvement in study interactions focusing on appreciative inquires and co-learning, together with university-based researchers, may have facilitated their way to the university in a city close to where they lived and worked. The Seniors did not get any reward other than the interactive elements of participation per se. Previous research [45] has highlighted questions concerning the appropriate amount and type of involvement, for example, and ways of recognition and monetary rewards for involvement of older people in research, which is worthy of consideration. In the current study, a main motivator for older peoples’ participation was articulated, in that one of the Seniors took it on herself to become a ‘spokesperson’ and explained why the involvement in a future-oriented project including IT-based solutions, like the eBalance project, was valued:

*“We older people do not want to be left behind”*.

## 5. Conclusions 

Our results indicate that the combination of PAAR methodology accompanied by NPT-inspired appreciative inquiries may be used as a rewarding research approach for user participation and co-learning in studies directed towards the development of electronic health (e-health) services for older adults. The initial phase of the eBalance project described here can be viewed as a first and promising feasibility study [46] focusing on the methodology, which should be further developed and applied in forthcoming phases of this project, aiming at technology and service development, testing, and implementation.

## Figures and Tables

**Table 1:** Overview of participants including their motives for participation.

Senior Citizens	Women/Men	Motives for Participation * (*n*)
	7/1	Experience of balance problems due to dizziness, Parkinson’s disease, or stroke (5)
		Experience of previous falls (2)
		Long history of exercising (1)
		Important with exercise support as the County Council has reduced resources (1)
**Rehabilitation Staff**		
	4/0	Area of interest (2)
		Can be useful in regular work (1)
		Development of own knowledge (1)
**Research Coordinators**		
	4/2	Research interests:
		Physical activity and health of older people (1)
		Geriatric care and rehabilitation (2)
		Balance, dizziness, and balance exercise (2)
		Human-computer interaction (1)
		Movement analysis (2)

* Answers from 6 Seniors Citizens, all 4 Rehabilitation Staff, and all 6 Research Coordinators. Some motives were given from more than one participant.

**Table 2:** Overview of the recruitment process of the participating user groups; Senior Citizens and Rehabilitation Staff, respectively.

	Interaction	Senior Citizens	Rehabilitation Staff
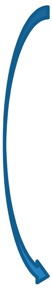	**First contact** 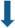	Asked if interested in participation at a Senior Health Convention. Contact details collected. All showing interest in participation invited to an information meeting.	Cooperation contract with the head of the community care of older people. Information to managers of rehabilitation, which asked for participation interest and approved participation during working hours. Invitation to an information meeting for all Staff showing interest in participation.
	**Information meetings** 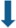	Information about the study for Seniors at the local health centre, including the opportunity to sign the list of interest for participation.	Information about the study for Staff at the local health centre, including the opportunity to sign the list of interest for participation.
	**Leaflet** 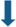	Study information for Seniors distributed at the information meeting, including a question for participation and contact details of the study coordinator.	Study information for Staff distributed at the information meeting, including a question for participation and contact details of the study coordinator.
	**List of Interest** 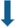	Opportunity to sign the list of interest, including giving motive for participation, at the information meeting or later through contact with the study coordinator.	Opportunity to sign the list of interest, including giving motive for participation, at the information meeting or later through contact with the study coordinator.
	**Invitation** 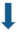	All 8 who met the inclusion criteria * were invited to participate.	All 4 who met the inclusion criteria ** were invited to participate.
	**Informed Consent**	Informed consent according to the Swedish regulations of ethical vetting of research on humans was given at the first workshop.	

* Inclusion criteria for Seniors = Independence in personal activities of daily living, access to and basic knowledge of a computer; ** Inclusion criteria for Staff = Currently working with the rehabilitation of older people.

**Table 3:** Overview of the user-interactions by workshops 1–5 (grey shading), between workshop home works 1–3 and the shared report, and related co-learning initiatives.

Interaction	Duration	Theme	Activity	PAAR-Type Reflective Inquiry
**Work shop 1**	1 day	Project Interaction	**Interaction task**: Pair-wise interview and presentation of participants **Presentation**: About this project and its interactive methodology, including group discussions Exergame * group work: Introduction, demonstration, and testing **Summarising reflections** in the whole group **Presentation**: Activity and health among older people and **Homework** **introduction**	Development of an appreciative gaze Reframing study aims Sharing new experiences and joint development of experienced-based knowledge Re-framing lived experiences Knowledge sharing to re-frame lived experiences and provide a basis for co-learning
**Home Work 1**		Meaningful activities in daily life?	**List and reflect** upon the question within 5 days, between WS1 and WS2	Appreciative knowledge-sharing to generate shared understanding and meaning, and co-learning
**Work shop 2**	2 h	Fall-prevention and IT-based support	**Presentation of project aims** **Homework follow-up** **Presentation**: Fall-prevention supported by IT **Group discussions** (NPT based questions **) **Summarising reflections** in whole group	Reframing study aims to generate shared understanding and meaning around them See above Knowledge sharing to generate shared understanding and meaning and co-learning Building practical wisdom
**Work shop 3**	2 h	Balance exercise and IT-support	**Presentation and testing**: Balance exercise **Exergame group work**, focusing on effective balance training **Reflection**: Think-peer-share **Homework introduction**	Knowledge-sharing to re-frame lived experiences and provide a basis for co-learning Sharing new experiences and joint development of experienced-based knowledge and practical wisdom
**Home Work 2**	5 days	Views on IT-based support for balance exercise?	**List and reflect** upon the theme (question) within 5 days, between WS3 and WS4	Knowledge sharing to generate shared understanding and meaning and co-learning as a base for building practical wisdom
**Work shop 4**	1 day	User-needs and design requests	**Presentation of project aims** **Movies**: Fall-prevention in the home and Balance exercise **Reflection**: Think-peer-share **Exercise-brake** for improved balance **Group-reflexions**: Factors of importance for project interaction (NPT based questions **) followed by **Whole-group reflection** of the above, including ranking	Generate shared understanding and meaning around the study aims Sharing new experiences and joint development of experienced-based knowledge and practical wisdom Same as above Appreciative knowledge sharing to generate shared understanding and meaning “Demonstrating achievement”—initiation
**Home Work 3**	3 weeks	Views on project interaction?	**List and reflect** upon the theme (question) within 5 days, between WS4 and WS5	Appreciative knowledge sharing for building practical wisdom and demonstrating achievement
**Work shop 5**	2.5 h	What we have learned	**Presentation of a summary of achievements** **Reflection**: Think-peer-share **Summarising reflections** in the whole group	Building practical wisdom to demonstrate achievements
**Shared Study report**		Our interactive work and what we together achieved	**Sharing drafts of the report** between participants **Considering feedback** from all participants **Sharing the final printed report** between all participants	Demonstrating achievements as basis for moving forward

* Wii and Prime Sense respectively; ** NPT based questions: see Appendix A; NPT: normalization process theory; PAAR: participatory and appreciative action and reflection; WS: work shop; IT: information technology.

## Appendix A

### ***Examples of Probing Questions Used for Reflections on Implementation Potential and Study*** ***Cooperation***

Questions inspired by the Normalization Process Theory [33] for reflections on important factors/requirements for (A) A high degree of implementation potential of fall-prevention exergames, and (B) A fruitful participant-interaction in the eBalance study. Section 1, Section 2, Section 3 and Section 4 refer to mechanism/essential work required for normalization according to the NPT:

(1) Coherence/Sense-making work, (2) Cognitive participation/Relational work, (3) Collective Action/Operational work, and (4) Reflexive Monitoring/Appraisal work.

(A) ***What is important for you and/or persons you work with, to …***
  A: 1
  Be ready to become engaged and test?
  Use it, even when you face uphill struggles and/or stress?
  Use it on a regular basis?
  Use it on a long-term basis as part of your activities in daily life/work-life?
  A: 2
  Experience support for the use of exergames?
  ○ Which person(s) near you (in your organization) need to be involved and support your use?
  ○ What instructions are needed?
  ○ What kind of feed-back is needed?
  ○ What kind of supportive communication is needed for discussions, advise and answering questions etc.?
  A: 3
  Judge it is secure enough to use?
  Experience that it is suitable to use?
  A: 4
  Be convinced that using it gives good effect?
(B) ***What is important for you to …***
  B: 1
  Be ready to be involved in the interaction?
  Continue participation?
  B: 2
  Get the opportunity to speak and be heard?
  B: 3
  Interact to facilitate sharing of experiences and knowledge?
  B: 4
  Improve the interaction?
